# One-Pot Synthesis and Characterization of CuCrS_2_/ZnS Core/Shell Quantum Dots as New Blue-Emitting Sources

**DOI:** 10.3390/ma16020762

**Published:** 2023-01-12

**Authors:** Ho-Kyung Lee, Ye-Jun Ban, Hyun-Jong Lee, Ji-Hyeon Kim, Sang-Joon Park

**Affiliations:** Department of Chemical and Biological Engineering, Gachon University, 1342, Seongnam Street, Seongnam City 13120, Republic of Korea

**Keywords:** CuCrS_2_, quantum dots, photoluminescence, blue emission

## Abstract

In this paper, we introduce a new blue-emitting material, CuCrS_2_/ZnS QDs (CCS QDs). To obtain bright and stable photoluminescent probes, we prepared a core/shell structure; the synthesis was conducted in a one-pot system, using 1-dodecanethiol as a sulfur source and co-ligand. The CCS QDs exhibited a semi-spherical colloidal nanocrystalline shape with an average diameter of 9.0 nm and ZnS shell thickness of 1.6 nm. A maximum photoluminescence emission peak (PL max) was observed at 465 nm with an excitation wavelength of 400 nm and PLQY was 5% at an initial [Cr^3+^]/[Cu^+^] molar ratio of one in the core synthesis. With an off-stoichiometric modification for band gap engineering, the CCS QDs exhibited slightly blue-shifted PL emission spectra and PLQY was 10% with an increase in initial molar ratio of 2.0 (462 nm PL max). However, when the initial molar ratio exceeded two, the CCS QDs exhibited a lower photoluminescence quantum yield of 4.5% with 461 nm of PL max at the initial molar ratio of four due to the formation of non-emissive Cr_2_S_3_ nanoflakes.

## 1. Introduction

Colloidal semiconductor nanocrystals, also called quantum dots (QDs), have been extensively investigated over the past three decades since the report on the synthesis of high-quality colloidal CdE (E = S, Se, and Te) in 1993 [1]. Due to the quantum confinement effect, the fluorescence wavelength of QDs can be tuned simply through the control of their size, shape, and composition. Although many studies have reported cadmium-based QDs due to their high quantum yield (QY) and full color tunability from blue to red [2], recently, Cd-free QDs are an emerging research area due to their inherent toxicity [3]. To apply the QDs to optoelectronic devices and biological area, the QDs should be less toxic and exhibit high performance such as high QY and stability.

Among QDs with various compositions, copper-based ternary QDs can be alternative sources for Cd-based QDs because they are less toxic and provide prominent optical properties. In particular, CuInS_2_ (CIS) QDs have been extensively investigated under bio-labelling [4], bio-sensing [5,6,7], gas sensors [8], light-emitting diodes (LEDs) [9], and solar cells [10]. They exhibit a high QY with the introduction of ZnS shells for surface passivation as high as over 50–80%, and the emission color can be tuned from green to near-infrared by controlling the size and Cu/In ratio [11,12,13]. However, because CIS QDs cannot be applied for the blue emission region of display applications, other alternatives for blue-emissive QDs with high QY and stability need to be investigated.

Several studies have been published related to blue-emissive QDs synthesis and characterization. For instance, Lesnyak et al. reported a method of blue-emitting ZnSe_1-x_Te_x_ alloy nanocrystals in an aqueous condition with a maximum photoluminescence (PL) wavelength at 425 nm and PL quantum efficiency (PLQE) of 20% [14]. In addition, Gao et al. prepared bulk-like ZnSe QDs as blue LEDs with a maximum PL peak at 446 nm, and PL quantum yield (PLQY) of ~100%, and external quantum efficiency (EQE) of 12.2% [15]. Additionally, Ji et al. fabricated strontium-doped CsPbCl_3_ superlattices for ultra-stable violet-emissive perovskite QDs (PQDs) with high PLQY of 82.4% [16]. However, their work required a significantly complex preparation process, such as various state precursors (solid and gas states), or an extremely careful step-by-step protocol for uniform and high-quality colloidal QDs. Accordingly, for commercialization, a simpler and more economic method for synthesis should be developed [17].

CuCrS_2_ is a Cu-based p-type semiconductor with optical band gap of approximately 2.48 eV. CuCrS_2_ has been frequently investigated in the thermoelectric field [18]; however, it has not been synthesized at nanoscales or as QDs for fluorescent probes. In this study, we prepared new blue-emitting CuCrS_2_/ZnS (CCS) QDs through a simple one-pot heating method with 1-dodecanethiol as a sulfur precursor and co-ligand. For band gap engineering, we used the off-stoichiometric modification method by varying the molar ratio of [Cr^3+^]/[Cu^+^] to induce blue shifting of the PL emission peak.

## 2. Experimental Section

### 2.1. Chemicals

Copper(I) acetate (Cu(OAc), 97%), chromium(III) nitrate nonahydrate (Cr(NO_3_)_3_·9H_2_O, 99%), zinc(II) acetate monohydrate (Zn(OAc)_2_·H_2_O, 99%), oleylamine (OAm, technical grade, 70%), 1-dodecanethiol (DDT, ≥98%), octadecene (ODE, technical grade, 90%), and chloroform anhydrous (99%) were purchased from Sigma Aldrich. Toluene (99%), acetone (99%), and ethyl acetate (99%) were purchased from Daejung Chemicals and Metals; all the chemicals were used without any further purification. 

### 2.2. Characterization

For the morphology characterization, a high-resolution transmission electron microscope (HR-TEM, Tecnai, FEI) was employed, with an accelerated voltage of 300 kV and resolution of 1.4 Å. To characterize the composition and crystal structure of synthesized QDs, a high-resolution X-ray photoelectron spectroscope (HR-XPS, Nexa, Thermofisher, Waltham, MA, USA) and high-resolution X-ray diffractometer (HR-XRD, Smartlab, Rigaku) with Al Kα X-ray and 3 kW Cu X-ray sources were employed, respectively. To characterize the optical properties of QDs, UV/Vis/NIR spectrophotometry (UV-Vis, V-770, Jasco) and spectrofluorometry (PL, Nanolog, Horiba) were utilized. In addition, fluorescence lifetime decay was measured with a time-resolved photoluminescence spectroscope (TRPL, Easylife, PTI).

### 2.3. Synthesis of CuCrS_2_/ZnS QDs in the One-Pot System

To synthesize CuCrS_2_ cores with a [Cr^3+^]/[Cu^+^] molar ratio of 1.0, 20 mL of ODE, 5 mL of OAm (~10 mmol), 0.375 mmol of Cu(OAc), and 0.375 mmol Cr(NO_3_)_3_·9H_2_O were mixed in a 100 mL three-neck round-bottomed flask; then, the mixture was degassed at 120 °C for 10 min. After degassing, 5 mL of DDT (~20 mmol) was added to the solution, which was then heated to 230 °C. Thereafter, it was stored for 1 h to form monomers and proceed with nucleation. After core synthesis, the solution was cooled down to room temperature; for a simultaneous ZnS shelling process, 4 mmol of Zn(OAc)_2_·H_2_O and 5 mL of OAm were added to the mixture and it was heated again to 120 °C for further degassing. Subsequently, 1 mL of DDT (~4 mmol) was injected, followed by heating up to 240 °C. After heating, the mixture was maintained at that temperature for 15 min for ZnS shelling onto the CuCrS_2_ core. For the purification step of the synthesized QDs, a small amount of toluene was added into the flask to dissolve the prepared QDs, and they were centrifuged at 5000 rpm for 5 min to remove larger particles and undesirable side-products. After removing by-products, they were precipitated by introducing an excess amount of acetone and performing centrifugation. After the QDs were precipitated, they were washed using the precipitation and re-dispersion method. Briefly, they were re-dispersed into a small amount of toluene, followed by precipitation with the addition of an excess amount of ethyl acetate. Subsequently, they were centrifuged at 5000 rpm for 5 min. This process was conducted one more time. After QDs were washed, they were dried overnight at 60 °C in a pre-heated vacuum oven. To compare the composition effect to the band gap of the QDs, we conducted the same process with only a change in the molar ratio of [Cr^3+^]/[Cu^+^], with values of 2:1 and 4:1. The prepared QDs with [Cr^3+^]/[Cu^+^] molar ratios of 1.0, 2.0, and 4.0 are denoted as 1.0 CCS, 2.0 CCS, and 4.0 CCS QDs, respectively. 

## 3. Results and Discussion 

### 3.1. Morphology and Crystal Structure of CuCrS_2_/ZnS QDs 

**Figure 1:**
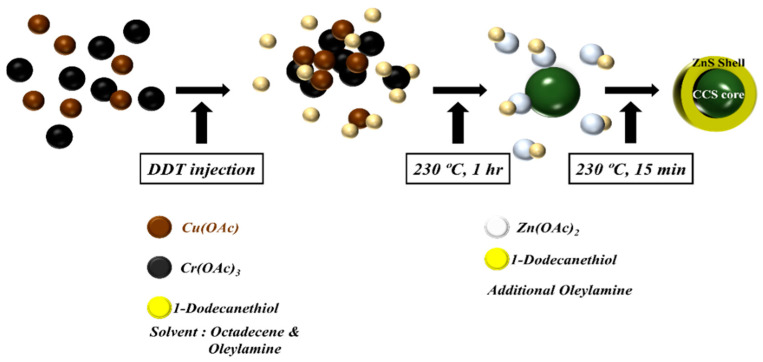
Schematic diagram of CCS QD synthesis.

**Figure 2:**
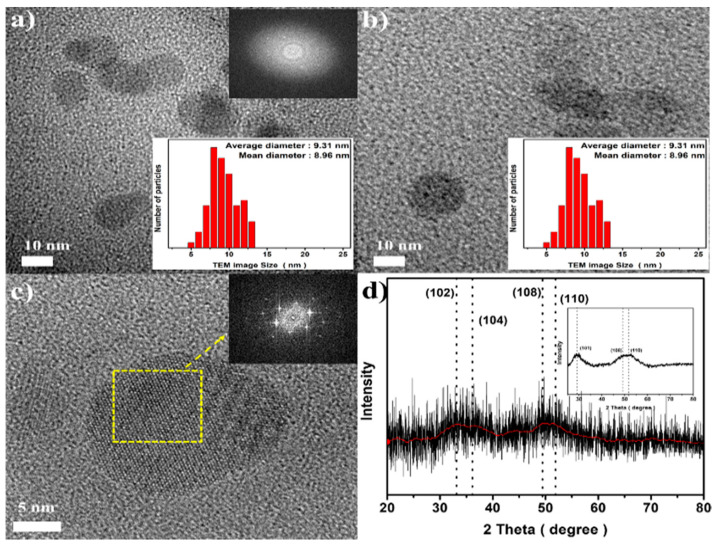
TEM images of (**a**) pure CuCrS_2_ core and (**b**) 1.0 CCS QDs. (**c**) FFT image containing the HR-TEM analysis result of 1.0 CCS QDs and (**d**) thin film XRD pattern of the CuCrS_2_ core and powder XRD pattern of 1.0 CCS QDs (interpolation).

CCS QDs were synthesized in an organic solvent via a simple heating process. Figure 1 shows a schematic diagram of CCS QDs synthesis. TEM analysis was conducted to investigate their morphological structure and the particle size. As shown in Figure 2a,b, the pure CuCrS_2_ nanocrystals and 1.0 CCS QDs exhibited an approximate spherical shape with mean diameters of 8.96 and 12.12 nm, respectively, and the ZnS shell thickness was confirmed to be approximately 1.6 nm by comparing the core and core/shell mean diameters. We clearly observed the lattice structure of the nanocrystals, showing that the 1.0 CCS QDs had high crystallinity (Figure 2c).

In addition, to analyze the crystal structure of the CCS QDs, we employed thin film and powder XRD techniques (Figure 2d). The XRD pattern of CuCrS_2_ core showed weak and broad peaks at 33.13°, 36.17°, 49.46°, and 51.89° indicating the (102), (104), (108), and (110) phases of hexagonal CuCrS_2_, respectively [19]. The absence of a (101) phase at around 30° was probably attributed to the partial release of Cu atoms at the washing step for XRD analysis. On the other hand, when the ZnS shell was coated onto the CuCrS_2_ core (Figure 1d interpolation graph), the (101) phase was observed, and every peak shifted slightly toward the smaller 2θ because the lattice parameter (a = 5.345 Å) of ZnS with a zinc blended structure is larger than that (a = 3.483 Å) of CuCrS_2_ [20]. To characterize the elemental composition and chemical oxidation state of the CCS QDs, we conducted XPS analysis for the pure CuCrS_2_ core and 1.0 CCS QDs. 

**Figure 3:**
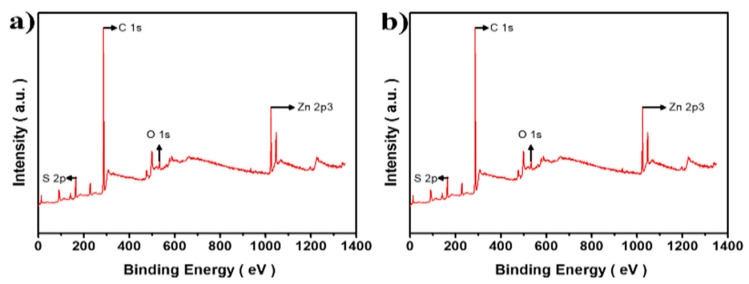
XPS spectra comparison of (**a**) pure CuCrS_2_ core and (**b**) 1.0 CCS QDs.

Figure 3 shows the XPS results of the pure CuCrS_2_ core and 1.0 CCS QDs. As shown in Figure 3b, which depicts the result of the CCS QDs XPS survey analysis, the Zn 2p peak was clearly observed with the disappearance of Cu 2p and Cr 2p peaks, which indicated the successful ZnS shell coating onto the CuCrS_2_ core. The N 1s peak in Figure 2a resulted from unwashed OAm on the surface of the CuCrS_2_ core. For deeper evaluation, high-resolution XPS analysis was also conducted.

**Figure 4:**
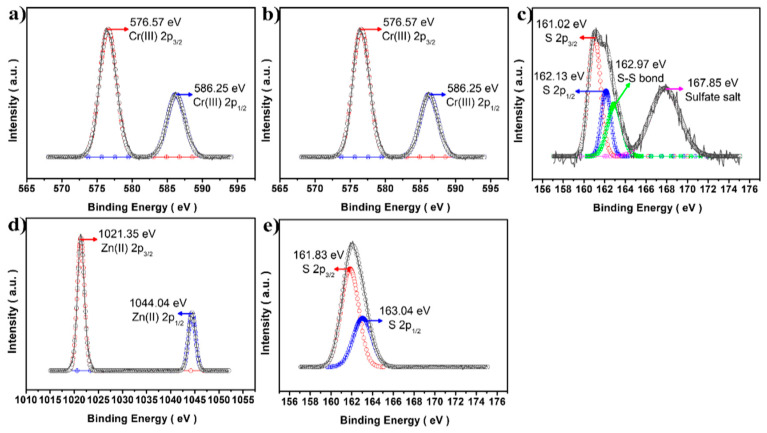
XPS analysis of the CuCrS_2_ core and CCS QDs. (**a**) Cu 2p, (**b**) Cr 2p, (**c**) S 2p spectra from the CuCrS_2_ core, and (**d**) Zn 2p, (**e**) S 2p from the 1.0 CCS QDs.

Figure 4a,b depicts the clear Cu(I) 2p_3/2_ and 2p_1/2_ peaks at 932.02 and 951.87 eV [21] and Cr(III) 2p_3/2_ and 2p_1/2_ at 576.57 and 586.25 eV [22], respectively. In addition, the CuCrS_2_ QDs core did not provide any unwanted state, such as Cu(II) or Cr(VI). Moreover, after the ZnS shell was formed, Zn 2p and S 2p spectra exhibited reasonably good spin–orbital coupling, indicating successful ZnS shell coating onto the CuCrS_2_ core. Interestingly, as shown in Figure 4c, some undesirable peaks appeared at 162.97 and 167.85 eV, indicating a S–S bond [23] and sulfate salt (SO_4_^2−^) state [24], in S 2p of the CuCrS_2_ core, respectively. The SO_4_^2−^ salt peaks might be attributed to the oxidizing activity of nitrate salts in Cr(NO_3_)_3_ with DDT, as a sulfur source, which probably results in the formation of SO_4_^2−^ in the CuCrS_2_ core level [25]. As proof, when the ZnS shell was coated, undesired peaks did not appear in the S 2p survey, which indicated that no other states were formed when nitrate salt was not present in the ZnS shell coating.

### 3.2. Band Gap Engineering of CuCrS_2_/ZnS QDs

To prepare the true-blue-emissive probe, we conducted band gap engineering of CCS QDs using a simple stoichiometric modification method by varying the molar ratio of [Cr^3+^]/[Cu^+^]. Then, the results were analyzed with UV–Vis and PL spectrometers. It is well known that the band gap of CIS QDs is significantly affected by the stoichiometry of the metal ions because the valence band (VB) of QDs is related to the copper vacancy level [26]. When copper vacancy decreases (Cu-deficient state CIS QDs), the ground state of VB of QDs is lowered due to the decrease in the Cu vacancy level, causing the band gap to broaden or increase [27,28]. Therefore, we employed this strategy to prepare the true-blue-emissive CCS QDs. Interestingly, the pure CuCrS_2_ exhibited a less- or non-emissive optical property after washing. Therefore, every subsequent experimental comparison was conducted through a ZnS shelling process with the same protocol. The optical band gap of prepared samples was calculated using the following Tauc plot equation with the direct transition method.

(1) $$E={\left(ahv\right)}^{1/2}$$

where *E* is the optical band gap of samples, *α* is an absorption coefficient of each sample, and *hv* is the photon energy. Then, relative PLQY was calculated using the Stern–Volmer equation:

(2) $$Q_{f}=Q_{r}\frac{I_{f}A_{r}}{I_{r}A_{f}}$$

where *Q* is the quantum yield, *I* is the integration of PL emission spectrum, and *A* is the absorbance of materials. The subscript *r* and *f* refer to the reference material and sample, respectively.

**Figure 5:**
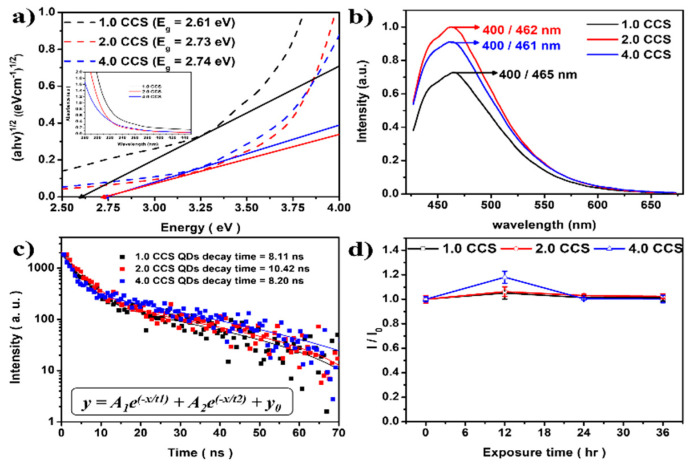
(**a**) Tauc plot result with UV–Vis spectra (intrapolation) and (**b**) PL spectrometer result of 1.0 CCS (black line), 2.0 CCS (red line), and 4.0 CCS (blue line) QDs. (**c**) Time-resolved PL lifetime decay curve, and (**d**) time-dependent photostability result under 365 nm irradiation.

To characterize the optical properties of the prepared CCS QDs, we conducted UV–Vis and PL measurements. As shown in Figure 5a, the optical band gap of the CCS QDs increased with the increase in the molar ratio of [Cr^3+^]/[Cu^+^] (2.61 eV for 1.0 CCS to 2.74 eV for 4.0 CCS). However, in the PL spectra (Figure 5b), a negligible blue shift in the maximum PL emission peak was observed, from 465 nm (1.0 CCS QDs) to 462 nm (2.0 CCS QDs) and 461 nm (4.0 CCS QDs). In Table 1, the optical properties of CCS QDs at different [Cr^3+^]/[Cu^+^] molar ratios are given. The 2.0 CCS QDs showed the highest PLQY, but further increase in molar ratio of [Cr^3+^]/[Cu^+^] caused a drastic decrease in the PLQY with a negligible blue shift. Fluorescence lifetime decay information is also presented in Table 1. The lifetime decay was fitted with a bi-exponential decay function (Figure 5c), and the decay times were obtained as 8.11 ns, 10.42 ns, and 8.2 ns for 1.0, 2.0, and 4.0 CCS QDs (Figure 5c), respectively. In addition, the photostability of CCS QDs were evaluated; it was found that CCS QDs showed good photostability under 365 nm light irradiation (Figure 5d), indicating that the core was well passivated by the ZnS shell.

**Figure 6:**
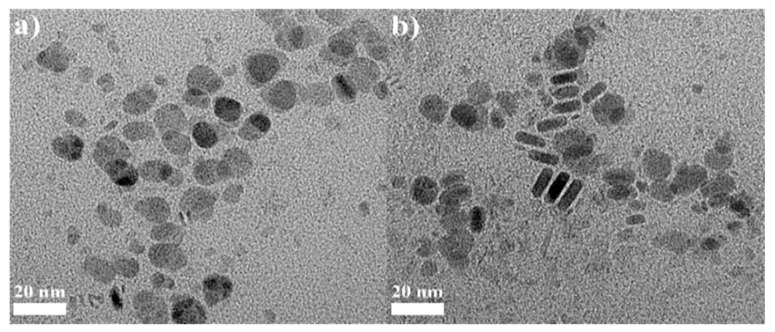
TEM images of the CuCrS_2_ core at Cr^3+^ ratios of (**a**) 2.0 and (**b**) 4.0.

Figure 6 shows results of the TEM analysis of the 2.0 and 4.0 CuCrS_2_ core. As shown in Figure 6a,b, some flake-shaped nanocrystals were observed. CuCrS_2_ consists of a series of alternating S–Cr–S triple layers perpendicular to the hexagonal *c*-axis in addition to an interlayer of copper atoms. S atoms form a distorted cubic close packing, whereas Cr atoms occupy tetragonal sites between the layers [29]. When the CuCrS_2_ core is synthesized, if the Cr^3+^ concentration is increased, *c*-axis-aligned Cr_2_S_3_ intermediates can be formed more easily, and these intermediates might undergo crystal growth during the synthesis, exhibiting a nanoflake shape. Therefore, more Cr^3+^ will cause unnecessary Cr_2_S_3_ nanoflakes. Without a ZnS shelling process, the copper atoms might be easily removed when washing, resulting in less- or non-emissive nanocrystals. This could be observed after synthesis of the CuCrS_2_ core without the ZnS shell. In this step, the unstable Cu atoms were washed out with the solvent and green precipitates were observed. Furthermore, CuCrS_2_ nanocrystals with [Cr^3+^]/[Cu^+^] ratios of 2.0 and 4.0 were investigated using high-resolution XPS analysis to compare the impurity peak intensities, such as SO_4_^2−^ in the CuCrS_2_ QDs core level.

**Figure 7:**
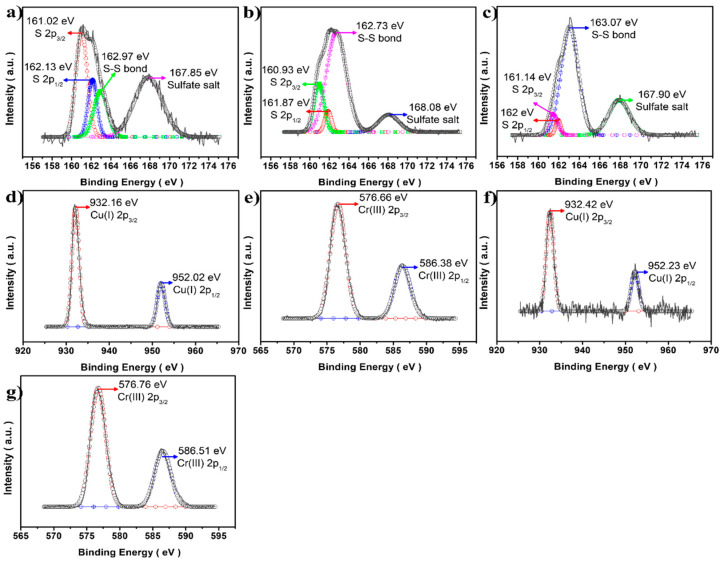
S 2p XPS comparison of the CuCrS_2_ core nanocrystals at different [Cr^3+^]/[Cu^+^] molar ratios. (**a**) 1.0, (**b**) 2.0, and (**c**) 4.0 ratios of CuCrS_2_. Cu 2p and Cr 2p spectra of (**d**,**e**) 2.0 CuCrS_2_ QDs and (**f**,**g**) 4.0 CuCrS_2_ QDs.

Figure 7 shows the XPS results for CuCrS_2_ core nanocrystals at different [Cr^3+^]/[Cu^+^] molar ratios. The Cu(I) 2p and Cr(III) 2p XPS spectra exhibited no meaningful change with increases in Cr^3+^ concentration (Figure 7d–g). The only difference was observed in the S 2p results. With increase in the Cr^3+^ molar ratio, the unwanted peak intensity at approximately 163 eV was also stronger in the S 2p analysis (Figure 7a–c), indicating that the disulfide bond formation might be improved [30,31]. The S 2p spin–orbital couples (S 2p_1/2_ and S 2p_3/2_) showed good agreement with a peak area ratio of approximately 2.0, indicating a typical S 2p orbital state [32]. This phenomenon probably resulted from Cr_2_S_3_ crystal growth with stacking, forming the nanoflakes. The increased Cr^3+^ concentration might accelerate the kinetics of Cr_2_S_3_ monomers, forming needless nanoflakes, as observed in the TEM analysis results (Figure 6). Due to these factors, the CCS QDs with high Cr^3+^ precursors exhibited a lower PLQY with a negligible blue shift.

## 4. Conclusions

We have introduced new blue-light-emissive CuCrS_2_/ZnS (CCS) quantum dots (QDs), which yielded a mean diameter of 12.1 nm with a ZnS shell with 1.6 nm thickness. The optical band gap and PLQY were confirmed to be 2.61 eV and 5% with 400/465 nm PLE/PL max at the initial molar ratio [Cr^3+^]/[Cu^+^] of 1.0, respectively. For obtaining true-blue-emissive QDs, the stoichiometric modification method was employed; it was found that the PL maximum emission peak was slightly blue-shifted from 465 nm to 462 nm with an increase in the initial molar ratio up to 2.0 (PL max 462 nm, PLQY ~ 10%). However, at the initial molar ratio of 4.0, i.e., greater than 2.0, the results showed that the impurity concentrations in the core level increased, such as SO_4_^2−^ and non-emissive Cr_2_S_3_ nanoflakes, which might cause the less blue shift and a decrease in the PLQY (PL max 461 nm, PLQY ~4.5%).

## Figures and Tables

**Table 1:** Optical properties of CCS QDs at different [Cr^3+^]/[Cu^+^] molar ratios.

Samples	[Cr^3+^]/[Cu^+^] Molar Ratio	PLE/PL	PL Lifetime Decay	Relative PLQY
1.0 CCS	0.375/0.375 (1.0)	400 nm/465 nm	8.11 ns	5%
2.0 CCS	0.5/0.25 (2.0)	400 nm/462 nm	10.42 ns	10%
4.0 CCS	0.6/0.15 (4.0)	400 nm/461 nm	8.2 ns	4.5%

## Data Availability

Not applicable.

## References

[1] Murray C.B., Norris D.J., Bawendi M.G. (1993). Synthesis and Characterization of Nearly Monodisperse Cde (E = S, Se, Te) Semiconductor Nanocrystallites. J. Am. Chem. Soc..

[2] Dabbousi B.O., RodriguezViejo J., Mikulec F.V., Heine J.R., Mattoussi H., Ober R., Jensen K.F., Bawendi M.G. (1997). (CdSe)ZnS core-shell quantum dots: Synthesis and characterization of a size series of highly luminescent nanocrystallites. J. Phys. Chem. B.

[3] Nam D.E., Song W.S., Yang H. (2011). Noninjection, one-pot synthesis of Cu-deficient CuInS2/ZnS core/shell quantum dots and their fluorescent properties. J. Colloid Interface Sci..

[4] Foda M.F., Huang L., Shao F., Han H.Y. (2014). Biocompatible and Highly Luminescent Near-Infrared CuInS2/ZnS Quantum Dots Embedded Silica Beads for Cancer Cell Imaging. ACS Appl. Mater. Interfaces.

[5] Liu S.Y., Shi F.P., Zhao X.J., Chen L., Su X.G. (2013). 3-Aminophenyl boronic acid-functionalized CuInS2 quantum dots as a near-infrared fluorescence probe for the determination of dopamine. Biosens. Bioelectron..

[6] Liu S.Y., Pang S., Na W.D., Su X.G. (2014). Near-infrared fluorescence probe for the determination of alkaline phosphatase. Biosens. Bioelectron..

[7] Zhang F.M., Ma P.Y., Deng X.Y., Sun Y., Wang X.H., Song D.Q. (2018). Enzymatic determination of uric acid using water-soluble CuInS/ZnS quantum dots as a fluorescent probe. Microchim. Acta.

[8] Zhang J., Hu D.E., Tian S.Q., Qin Z.Y., Zeng D.W., Xie C.S. (2018). CuInS2 QDs decorated ring-like NiO for significantly enhanced room-temperature NO2 sensing performances via effective interfacial charge transfer. Sens. Actuators B-Chem..

[9] Park S.H., Hong A., Kim J.H., Yang H., Lee K., Jang H.S. (2015). Highly Bright Yellow-Green-Emitting CuInS2 Colloidal Quantum Dots with Core/Shell/Shell Architecture for White Light-Emitting Diodes. ACS Appl. Mater. Interfaces.

[10] Ilaiyaraja P., Rakesh B., Das T.K., Mocherla P.S.V., Sudakar C. (2018). CuInS2 quantum dot sensitized solar cells with high V-OC approximate to 0.9 V achieved using microsphere-nanoparticulate TiO2 composite photoanode. Sol. Energy Mater. Sol. Cells.

[11] Song W.S., Yang H. (2012). Efficient White-Light-Emitting Diodes Fabricated from Highly Fluorescent Copper Indium Sulfide Core/Shell Quantum Dots. Chem. Mater..

[12] Uehara M., Watanabe K., Tajiri Y., Nakamura H., Maeda H. (2008). Synthesis of CuInS2 fluorescent nanocrystals and enhancement of fluorescence by controlling crystal defect. J. Chem. Phys..

[13] Yoon S.Y., Kim J.H., Jang E.P., Lee S.H., Jo D.Y., Kim Y., Do Y.R., Yang H. (2019). Systematic and Extensive Emission Tuning of Highly Efficient Cu-In-S-Based Quantum Dots from Visible to Near Infrared. Chem. Mater..

[14] Lesnyak V., Dubavik A., Plotnikov A., Gaponik N., Eychmuller A. (2010). One-step aqueous synthesis of blue-emitting glutathione-capped ZnSe1-xTex alloyed nanocrystals. Chem. Commun..

[15] Gao M., Yang H.W., Shen H.B., Zeng Z.P., Fan F.J., Tang B.B., Min J.J., Zhang Y., Hua Q.Z., Li L.S. (2021). Bulk-like ZnSe Quantum Dots Enabling Efficient Ultranarrow Blue Light-Emitting Diodes. Nano Lett..

[16] Ji Y.Q., Wang M.Q., Yang Z., Wang H., Padhiar M.A., Qiu H.W., Dang J.L., Miao Y.R., Zhou Y., Bhatti A.S. (2022). Strong violet emission from ultra-stable strontium-doped CsPbCl3 superlattices. Nanoscale.

[17] Lee W., Lee C., Kim B., Choi Y., Chae H. (2020). Synthesis of Blue-Emissive InP/GaP/ZnS Quantum Dots via Controlling the Reaction Kinetics of Shell Growth and Length of Capping Ligands. Nanomaterials.

[18] Tewari G.C., Tripathi T.S., Kumar P., Rastogi A.K., Pasha S.K., Gupta G. (2011). Increase in the Thermoelectric Efficiency of the Disordered Phase of Layered Antiferromagnetic CuCrS2. J. Electron. Mater..

[19] Korotaev E.V., Syrokvashin M.M., Filatova I.Y., Zvereva V.V. (2021). Magnetic Properties of Novel Layered Disulfides CuCr0.99Ln0.01S2 (Ln = La horizontal ellipsis Lu). Materials.

[20] Mantella V., Varandili S.B., Pankhurst J.R., Buonsanti R. (2020). Colloidal Synthesis of Cu-M-S (M = V, Cr, Mn) Nanocrystals by Tuning the Copper Precursor Reactivity. Chem. Mater..

[21] Xie B.B., Hu B.B., Jiang L.F., Li G., Du Z.L. (2015). The phase transformation of CuInS2 from chalcopyrite to wurtzite. Nanoscale Res. Lett..

[22] Chen Y.A., An D., Sun S.A., Gao J.Y., Qian L.P. (2018). Reduction and Removal of Chromium VI in Water by Powdered Activated. Carbon. Materials.

[23] Krylova V. (2013). Deposition and characterization of silver sulfide layers on the polypropylene film surface. Chemija.

[24] Lin X.Y., Liu R.T., Lin W., Li Z.Z., Xiang X., Ning L., Jie C. (2021). The synergistic effect of carbon materials on properties of copper-based friction materials. Ind. Lubr. Tribol..

[25] Fichtner T., Fischer A.R., Dornack C. (2021). Nitrate consumption by the oxidation of sulfides during an enhanced natural attenuation project at a contaminated site in Berlin, Germany. Environ. Sci. Eur..

[26] Morselli G., Villa M., Fermi A., Critchley K., Ceroni P. (2021). Luminescent copper indium sulfide (CIS) quantum dots for bioimaging applications. Nanoscale Horiz..

[27] Chen B.K., Zhong H.Z., Zhang W.Q., Tan Z.A., Li Y.F., Yu C.R., Zhai T.Y., Bando Y.S., Yang S.Y., Zou B.S. (2012). Highly Emissive and Color-Tunable CuInS2-Based Colloidal Semiconductor Nanocrystals: Off-Stoichiometry Effects and Improved Electroluminescence Performance. Adv. Funct. Mater..

[28] Chuang P.H., Lin C.C., Liu R.S. (2014). Emission-Tunable CuInS2/ZnS Quantum Dots: Structure, Optical Properties, and Application in White Light-Emitting Diodes with High Color Rendering Index. ACS Appl. Mater. Interfaces.

[29] Han C.G., Zhang B.P., Ge Z.H., Zhang L.J., Liu Y.C. (2013). Thermoelectric properties of p-type semiconductors copper chromium disulfide CuCrS2+x. J. Mater. Sci..

[30] Koroteev V.O., Bulushev D.A., Chuvilin A.L., Okotrub A.V., Bulusheva L.G. (2014). Nanometer-Sized MoS2 Clusters on Graphene Flakes for Catalytic Formic Acid Decomposition. ACS Catal..

[31] Koroteev V.O., Bulusheva L.G., Asanov I.P., Shlyakhova E.V., Vyalikh D.V., Okotrub A.V. (2011). Charge Transfer in the MoS2/Carbon Nanotube Composite. J. Phys. Chem. C.

[32] Turo M.J., Macdonald J.E. (2014). Crystal-Bound vs Surface-Bound Thiols on Nano crystals. ACS Nano.

